# The Elevational Distribution Patterns and Driving Factors of Plant Carbon Storage Across Different Functional Groups in Subalpine Grasslands of the Eastern Loess Plateau, China

**DOI:** 10.3390/plants15111696

**Published:** 2026-05-30

**Authors:** Manhou Xu, Jiaying Wang, Kunkun Wei, Xiuli Yu, Na Huo

**Affiliations:** 1 School of Geographic Sciences, Taiyuan Normal University, Jinzhong 030619, China; 15525052352@163.com (J.W.); dejb70110@gmail.com (K.W.); axiu985211@163.com (X.Y.); huonamail@163.com (N.H.); 2 Shanxi Key Laboratory of Earth Surface Processes and Resource Ecology Security in Fenhe River Basin, Taiyuan Normal University, Jinzhong 030619, China; 3 Institute of Carbon Neutrality, Taiyuan Normal University, Jinzhong 030619, China; 4 Key Laboratory of Restoration Ecology of Cold Area in Qinghai Province, Northwest Institute of Plateau Biology, Chinese Academy of Sciences, Xining 810008, China

**Keywords:** Loess Plateau, subalpine grassland, plant carbon storage, elevation, PLS-SEM (partial least squares structural equation model)

## Abstract

Subalpine grasslands (SGs) of the Loess Plateau in China play a crucial role in the global carbon cycle of terrestrial ecosystems. However, the distribution pattern of total carbon stores along an elevation gradient on the SG plants of the eastern plateau remains unclear. In this study, eight typical mountains with one well-developed SG being surveyed as plot for each mountain were selected along an elevation gradient from 1722 m to 2954 m on the east of the plateau. The vegetation area, hydrothermal factors, soil elements, and species composition were analyzed using methods of spatial analysis and a partial least squares structural equation model (PLS-SEM), and these were used to estimate the total carbon stores of different plant functional groups for the entire area of each SG. This study revealed the driving factors of the elevational pattern of plant carbon storage in the SGs. The entire plant carbon storage of the eight SGs was 35,880.98 Mg in total. In addition, the aboveground and belowground carbon storage values both exhibited U-shaped trends along the elevation gradient. Significant minimum values were observed at the mid-elevation regions, ranging from 2305 m to 2673 m. The plant carbon storage was predominantly allocated to the belowground portions (accounting for 72.3% of the total carbon storage), and this allocation strategy was more pronounced at both low- and high-elevation regions. The carbon storage proportion among the different plant functional groups was the largest for forbs (average in 2348.85 Mg, accounting for 52%), medium for sedges (average in 1982.81 Mg, accounting for 44%), and the smallest for grasses (average in 153.47 Mg, accounting for 4%). The plant species diversity promoted carbon accumulation in the sedges and forbs, while the soil total phosphorus exhibited an inhibitory effect. In the PLS-SEM, hydrothermal factors (total effect = −0.8107) and species diversity (total effect = 0.4969) were the primary drivers of the plant carbon storage elevational pattern in the SGs, while the soil properties (total effect = −0.3501) and biomass (total effect = 0.0697) effects did not reach statistical significances. Therefore, the plant carbon storage distribution pattern along the elevation gradient was driven by hydrothermal factors and species diversity on the SGs of the eastern plateau. The plants such as forbs and sedges might play more important roles in improving regional plant carbon storage in high-elevation grasslands, through interactions with hydrothermal factors.

## 1. Introduction

Anthropogenic greenhouse gas emissions have reached unprecedented levels over the past 800,000 years [1]. Global near-surface temperatures have exhibited sustained increases since the 19th century, with the 1983–2012 periods representing the warmest three-decade interval in the past eight centuries [2]. Since 2007, China has become the world’s largest emitter of energy-related CO_2_, and the country faces increasing pressure to reduce carbon emissions [3].

Grasslands are globally widespread and functionally vital terrestrial ecosystems that fulfill critical roles for long-term carbon sequestration, the maintenance of regional and global biodiversity, and climate change mitigation [4]. Grasslands are a globally significant carbon reservoir, with their carbon storage (namely, total stores for the entire area) estimated to be approximately 308 Pg [5]. This number represents approximately 34% of the carbon stored in global terrestrial ecosystems. China’s grasslands cover an area of approximately 400 million hectares, constituting 41.7% of the nation’s total terrestrial land area [6]. Their carbon storage is estimated to be approximately 29.1 Pg [7], accounting for roughly 10% of the global grassland carbon pool [8]. These numbers indicate that grassland ecosystems have a substantial carbon sequestration potential and perform critical functions in the achievement of regional and global carbon neutrality targets.

Grassland vegetation assimilates atmospheric CO_2_ into organic carbon through photosynthesis. This organic carbon is subsequently sequestered into the plant biomass. This process is a crucial pathway for atmospheric carbon influx into grassland ecosystems, providing the material and energy foundation for internal carbon cycling [9]. Current studies regarding the elevational pattern of carbon storage in grassland plants have revealed pronounced regional heterogeneity. For instance, in the alpine meadows of the Qinghai–Tibet Plateau, the plant carbon storage results were inconsistent, with some studies reporting both increasing [10] and decreasing [11] trends with elevation. Similarly, carbon storage in the mountain grasslands of the Yanqi Hola Mountain exhibited fluctuations along the elevational gradient [12]. The alpine herbaceous plants in the Ecuadorian Andes were shown to possess a declining pattern, with a sharp decrease in the carbon pool [13]. This has been more consistently observed in other high-elevation systems. For example, a significant reduction was found in the plant carbon storage in the tropical Páramo grasslands [14]. Conversely, temperate grassland studies on the Loess Plateau have documented a positive correlation between plant carbon storage and elevation [15]. These contrasting patterns underscore the joint regulation of the elevational carbon storage distribution in grassland plants due to the vegetation composition and local-to-regional climatic conditions.

Additionally, the carbon storage of grassland plants varies across different functional groups. In the subalpine grasslands (SGs) of Shanxi Province [16], the carbon storage among different plant functional groups were ranked as follows: sedges (1.32 Tg) > forbs (0.42 Tg) > grasses (0.40 Tg). Zhang et al. [17] further demonstrated that the carbon storage varied significantly across plant functional groups, and these groups exhibited distinct patterns of carbon storage along elevational gradients. In a recent study on carbon density (namely, the carbon storage per unit area), a consistent increase in the carbon density with elevation across multiple plant functional groups within the SGs was observed [16]. Conversely, Muhammad et al. [18] examined the temperate coniferous forests of Kalam, Pakistan, and they reported a decreasing trend in the plant carbon storage across functional groups with increasing elevation that peaked within the mid- to low-elevation zones. Therefore, under specific climatic and vegetation contexts, investigating the elevational patterns of carbon storage across plant functional groups is crucial to accurately predict the spatial heterogeneity of grassland carbon cycles and inform targeted ecological management strategies.

In addition to the documentation of spatial patterns, the driving factors of spatial variations in grassland plant carbon storage have become a focal point in ecological research. The carbon storage spatial distribution in grassland plants is regulated by biotic and abiotic factors. Species diversity is a biological factor that enhances the carbon sequestration potential through the promotion of community productivity [19], while plant biomass, which is the direct material basis of carbon storage, fundamentally determines its magnitude [9]. Climatic factors (e.g., precipitation and temperature) are abiotic drivers that fundamentally regulate plant growth and carbon accumulation processes. For example, increased precipitation enhances the primary productivity and carbon storage of grasslands in semi-arid regions [20]. Simultaneously, soil properties (e.g., texture, pH, and nutrient contents) are crucial for the regulation of grassland plant carbon storage [21]. Moreover, various management practices can significantly influence plant carbon storage [22,23,24]. Collectively, these studies on the driving factors have demonstrated that the carbon storage spatial distribution in grassland plants is shaped by the complex interactions of biotic and abiotic factors whose relative contributions may vary across plant functional groups [16]. Therefore, the elucidation of plant carbon storage spatial distribution characteristics and their governing factors across different functional groups in grasslands is crucial to accurately understand the spatial heterogeneity of regional carbon cycles and their responses to climate change.

SGs are vital grassland types that provide significant ecosystem services such as water conservation, climate regulation, and biodiversity maintenance [25]. SGs are a fragile ecological environment, but they do exhibit a substantial carbon sink potential [26]. Research has indicated that SGs harbor carbon storage as high as 18.37 Pg [7], with an estimated annual increase of 1.14 Tg occurring from 1982 to 2018 [27]. These findings demonstrated a steady enhancement in the carbon sequestration capacity of SGs. Moreover, the net ecosystem carbon exchange in SGs is primarily regulated by temperature [27], and this underscores their critical role in the carbon cycle under climate change. Loess Plateau SGs are characterized by distinctive regional attributes, notably their spatial continuity and pronounced ecological multifunctionality [28]. The eastern portion of the Loess Plateau lies at the transitional zone between China’s second and third topographic steps. This unique geographic setting and climatic conditions have fostered extensive SGs that cover approximately 353,000 hm^2^. Due to the implementation of ecological conservation and restoration measures in recent years [29,30,31], vegetation coverage in the SGs of the eastern plateau has increased; hence, their carbon sink potential has gradually become apparent [29,30]. Our previous research revealed that plant functional groups influence the spatial distribution of the carbon density on the SGs of the eastern Plateau [16]. However, for the total carbon stores of SGs on the eastern plateau, the distribution pattern of plant carbon storage for the entire area along an elevation gradient remains unclear [32]. Consequently, the total carbon stores of different plant functional groups in the entire area and the driving factors of the elevational plant carbon storage distribution pattern in this region are still unsolved. This limits the development of accurate assessments and precise management strategies for the grassland carbon sink potential on the Loess Plateau.

In this study, eight typical mountains with one well-developed SG being surveyed as plot for each mountain were selected along an elevation gradient (1722–2954 m) on the eastern plateau. Based on the total carbon stores for the entire area in each SG, we then explored the elevational pattern of the carbon storage in grassland plants and its driving factors. We tested the following two hypotheses: (1) carbon storage in SG plants on the eastern plateau exhibits a nonlinear distribution pattern along the elevation gradient, with this elevational spatial differentiation may being driven by the effects of hydrothermal conditions and species diversity or other bio- and abiotic factors; and (2) the carbon storage changes across different plant functional groups in this region, with forbs and sedges may playing a dominant role in carbon sequestration within communities. These findings will provide scientific support for carbon sink management on the plateau and promote the sustainable development of SG ecosystems at high-elevation areas.

## 2. Results

### 2.1. Plant Carbon Storage Accumulations for the Entire Area in Each SG

#### 2.1.1. Aboveground, Belowground, and Total Carbon Storage of SG Plants

The carbon storage data of the SG plants were highly variable (Table 1). The total carbon storage (TC) ranged between 1125.1 and 8174.53 Mg, the aboveground carbon storage (AGC) ranged between 323.54 and 2903.23 Mg, and the belowground carbon storage (BGC) ranged between 694.09 and 6596.10 Mg. All three components (TC, AGC, and BG) exhibited significant numerical variations. Among these, the AGC exhibited the highest coefficient of variation, followed by the BGC, and the TC showed the least fluctuation. This pattern indicated significant spatial heterogeneity in plant carbon storage across the SGs, with the aboveground carbon storage exhibiting a greater sensitivity to the prevailing cold climate conditions. The average belowground carbon storage (BGC, 3232.18 Mg) was approximately 2.5 times greater than the aboveground component (AGC, 1252.94 Mg), and it constituted 72.3% of the total carbon storage (TC, 4485.12 Mg). This allocation pattern suggested a preferential belowground partitioning of plant carbon under the prevailing cold climate conditions in high-elevation grasslands.

#### 2.1.2. Carbon Storage of the Different Functional Groups in SG Plants

The plant carbon storage exhibited distinct patterns across the functional groups (Table 2). Overall, the forb communities exhibited significantly higher aboveground, belowground, and total carbon storage than that of the grasses and sedges. The forbs community had the largest aboveground, belowground, and total carbon storage, amounting to 704.80 Mg, 1644.05 Mg, and 2348.85 Mg, respectively. Grasses had the lowest storage (e.g., total carbon: 153.47 Mg) with high data dispersion. In contrast, sedges and forbs achieved higher storage levels with more concentrated value distributions. These divergences likely stemmed from their contrasting biological traits, which led to significantly different responses to geographic environmental gradients.

**Figure 1:**
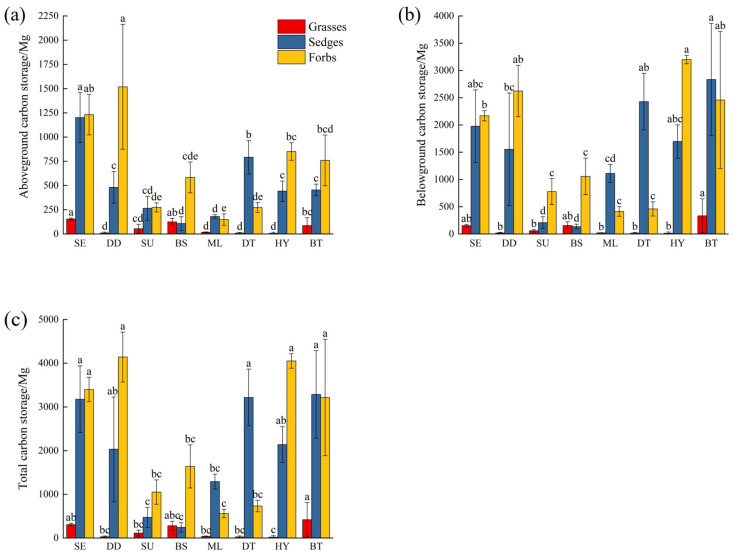
Carbon storage of plant functional groups with grasses, sedges, and forbs across the SGs: aboveground (**a**), belowground (**b**), and total (**c**). Values are presented as means ± S.D. (*n* = 20 quadrats per SG). The different lowercase letters above the error bars denote significant differences among the SGs within each functional group (one-way ANOVA, Tukey’s HSD test, *p* < 0.05). The F-values and degrees of freedom from the ANOVAs are shown in Supplementary Table S4. Abbreviations in x-axises are SE (Shengwangping), DD (Diandingshan), SU (Shunwangping), BS (Bashuigou), ML (Maluncaoyuan), DT (Dongtai), HY (Heyeping), and BT (Beitai). They are names of surveyed mountains in Table 3.

For the aboveground carbon storage (Figure 1a), the SE (Shengwangping) site had the highest storage for both the grasses (153.93 Mg) and sedges (1201.23 Mg), whereas the DD (Diandingshan) site supported the highest storage for forbs (1518.3 Mg). For the belowground carbon storage (Figure 1b), the BT (Beitai) site showed the highest storage for the grasses (332.32 Mg) and sedges (2833.4 Mg), whereas the highest storage for forbs (3199.93 Mg) was recorded at the HY (Heyeping) site. For the total carbon storage (Figure 1c), the storage varied markedly across sites for each functional group. The BT site had the highest grass carbon storage (418.57 Mg), which was significantly greater than that at the DD, SU (Shunwangping), ML (Maluncaoyuan), DT (Dongtai), and HY sites, with HY having the lowest value (19.77 Mg). Similarly, the sedge carbon storage was highest at BT (3287.79 Mg), and it significantly exceeded the levels at the SU, BS (Bashuigou), and ML sites. For forbs, the DD site had the highest carbon storage (4141.56 Mg), and it significantly surpassed those at the SU, BS, ML, and DT sites.

**Figure 2:**
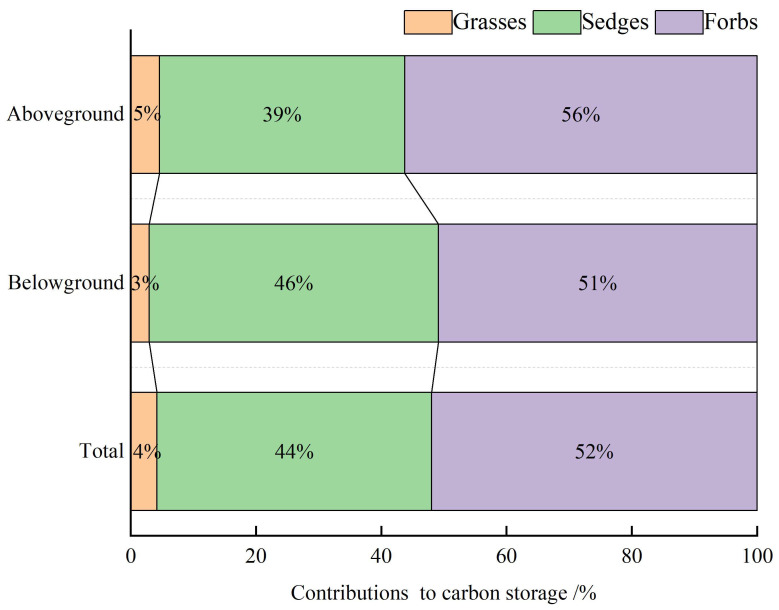
Proportional contributions of plant functional groups to community carbon storage: aboveground, belowground, and total. The stacked bars show the mean percentage of each group’s contribution to the aboveground, belowground, and total carbon pools. The percentages are derived from the mean carbon storage of each group across all SGs, with the quadrat number being 160 (i.e., 20 quadrats for each of the 8 SGs).

An analysis of the carbon storage composition revealed that forbs constituted the largest proportion (52%) and served as the primary contributor, sedges formed the secondary component (44%), while grasses accounted for a minor fraction (4%) (Figure 2). This revealed an overall pattern in the carbon storage allocation among the three functional groups that followed the order: forbs > sedges > grasses. Grasses and forbs showed similar allocation patterns of the carbon storage, with a relatively higher proportion allocated aboveground. In contrast, sedges showed a more pronounced belowground allocation. This result reflected the differential utilization of the aboveground and belowground niche spaces by distinct plant functional groups, which, in turn, drives adaptive divergence in their carbon allocation strategies.

**Table 3:** Geographic coordinates and mountain abbreviations for the eight surveyed SGs in the eastern Loess Plateau. The surveyed mountains were ordered by increased elevations and were all less than 3000 m; hence, the grasslands in these mountains belonged to the subalpine vegetation type. The number of plant species in each SG is the total surveyed in 2023 and 2024, with the repeated species only being counted once. The detained species with different functional groups of grasses, sedges, and forbs are shown in Supplementary Table S1.

Sample Number	Mountain Name	Elevation (m)	Longitude (°)	Latitude (°)	Number of Plant Species
1	Shengwangping (SE)	1722	112.21	35.53	23
2	Diandingshan (DD)	2282	113.97	39.85	23
3	Shunwangping (SU)	2305	111.96	35.42	25
4	Bashuigou (BS)	2486	111.42	37.83	13
5	Maluncaoyuan (ML)	2673	111.93	38.75	18
6	Dongtai (DT)	2724	113.66	39.04	21
7	Heyeping (HY)	2742	111.84	38.71	20
8	Beitai (BT)	2954	113.58	39.08	19

### 2.2. Elevational Distributions of Plant Carbon Storage for the Entire Area in Each SG

**Figure 3:**
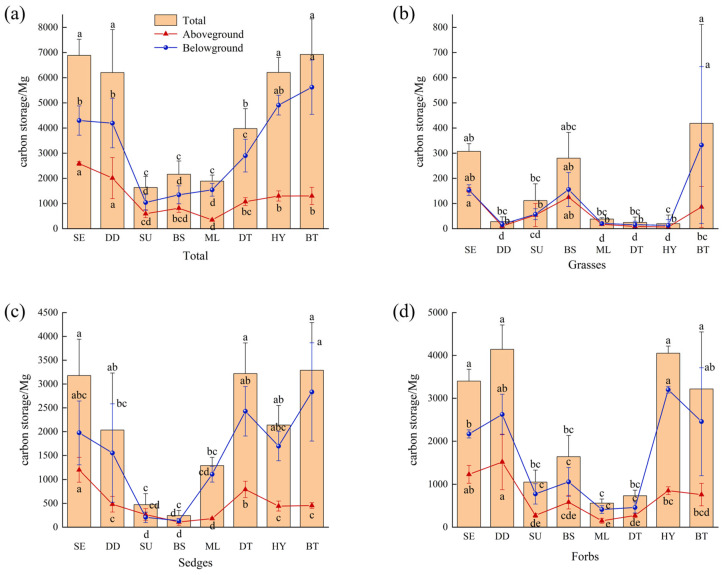
Elevational plant carbon storage patterns. From SE to BT, the elevation is increased, with elevations being listed in Table 3. (**a**) The total carbon storage across all functional groups. (**b**–**d**) The carbon storage for grasses (**b**), sedges (**c**), and forbs (**d**). Data are represented as the means ± S.D. (*n* = 20 quadrats per SG). The different lowercase letters indicate significant differences along the elevational gradient (one-way ANOVA, Tukey’s HSD test, *p* < 0.05). The F-values and degrees of freedom from the ANOVAs are shown in Supplementary Table S4. Abbreviations are the same as in Figure 1.

The aboveground, belowground, and total carbon storage of SG plants exhibited a U-shaped trend (with quadratic models being better among common curve estimations) along the elevational gradient (Figure 3 and Supplementary Table S6). The most distinct feature was a significant minimum that occurred within the mid-elevation zone (at approximately 2305–2673 m) (Figure 3a). This was accompanied by substantial variability across the full gradient. The aboveground and total carbon storage initially decreased and then increased with elevation. This resulted in a net significant decrease across the entire gradient (*p* < 0.001). The belowground carbon storage initially decreased and then increased with elevation. This resulted in a net significant increase across the entire gradient (*p* < 0.001).

The belowground carbon storage consistently exceeded the aboveground carbon storage. The allocation strategy between these pools varied systematically with elevation, and both the low- and high-elevation zones exhibited a pronounced bias toward belowground allocation, whereas the aboveground and belowground carbon storage difference was minimal in the mid-elevation zones. This shift reflected an adaptive response to the distinct environmental conditions that prevailed across the elevational gradient.

Across different functional groups, the grasses’ carbon storage (aboveground, belowground, and total) showed a significant declining trend with increasing elevation (*p* < 0.05), albeit with noticeable fluctuations along the gradient (Figure 3b). In contrast, the carbon storage of sedges (Figure 3c) and forbs (Figure 3d) followed a unimodal pattern that initially decreased and then increased with elevation. Despite this mid-elevation increase, a highly significant net decline was observed across the entire elevational gradient (*p* < 0.001).

### 2.3. Driving Factors of Plant Carbon Storage for the Entire Area in Each SG

#### 2.3.1. Correlations of Plant Carbon Storage with Biotic and Abiotic Factors

**Figure 4:**
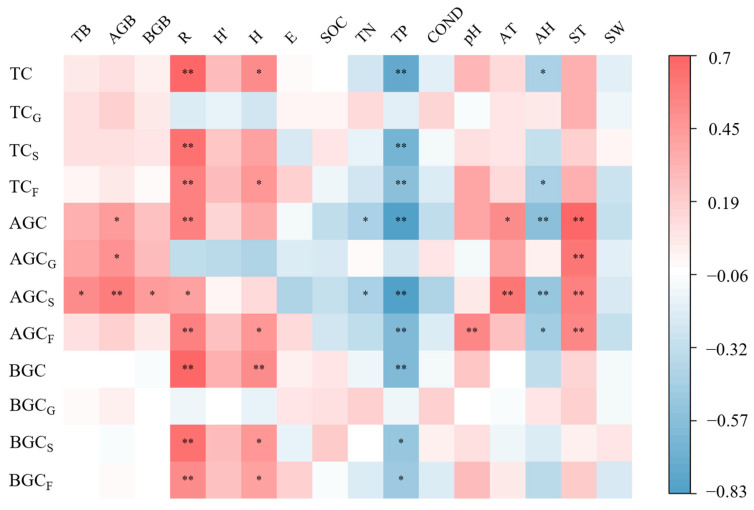
Heatmap of the correlations in the plant carbon storage with biotic and abiotic factors. The biotic factors included the total biomass (TB), the aboveground biomass (AGB), the belowground biomass (BGB), the Patrick richness (R), the Simpson index (H′), the Shannon index (H), and the Pielou index (E). The abiotic factors comprised the soil organic carbon (SOC), the total nitrogen (TN), the total phosphorus (TP), the electrical conductivity (COND), the power of hydrogen (pH), the air temperature (AT), the air humidity (AH), the soil temperature (ST), and the soil water content (SW). TC, AGC, and BGC were the total, aboveground, and belowground carbon storage, respectively. AGC_G_, AGC_S_, and AGC_F_ were the AGC for grasses, sedges, and forbs, respectively. BGC_G_, BGC_S_, and BGC_F_ were the BGC for grasses, sedges, and forbs, respectively. The values denote the Pearson’s correlation coefficients with color intensity being the strength and direction of the correlation (i.e., red: positive; blue: negative). The asterisks denote the significance levels (i.e., *: *p* < 0.05; **: *p* < 0.01). Data were obtained from 160 quadrats from all of the surveyed SGs. The specific r and *p* values are provided in Supplementary Table S5.

The total carbon storage of grasses (TC_G_) exhibited no significant correlation with any of the measured driving factors (Figure 4). Both TC and TC_F_ (total carbon storage of forbs) exhibited significant positive correlations with R (Patrick richness) and H (Shannon index) and significant negative correlations with TP (total phosphorus) and AH (air humidity). The total carbon storage of sedges (TC_S_) showed a significant positive correlation with R and a significant negative correlation with TP. These results suggested that for the total carbon storage, species diversity enhanced carbon sequestration in sedge and forb communities, whereas elevated TP and AH levels exhibited inhibitory effects.

The aboveground carbon storage in grasses (AGC_G_) showed significant positive correlations with the AGB (aboveground biomass) and ST (soil temperature) (Figure 4). Both AGC and AGC_S_ (aboveground carbon storage in sedges) exhibited significant positive correlations with the AGB, R, AT (air temperature), and ST and significant negative correlations with the TN (total nitrogen), TP, and AH. The aboveground carbon storage in forbs (AGC_F_) showed significant positive correlations with R, H, pH (power of hydrogen), and ST and significant negative correlations with TP and AH. This suggested that for the aboveground carbon storage, biological factors (e.g., biomass) and temperature promoted accumulation across the functional types, whereas elevated TP and AH levels inhibited accumulation, specifically in sedges and forbs.

The belowground carbon storage of grasses (BGC_G_) showed no significant correlation with any driving factors (Figure 4). BGC, BGC_S_ (belowground carbon storage of sedges), and BGC_F_ (belowground carbon storage of forbs) all exhibited significant positive correlations with R and H and significant negative correlations with TP. This result indicated that in the belowground portion, species diversity promoted carbon storage in sedges and forbs, while the TP content exerted an inhibitory effect.

#### 2.3.2. PLS-SEM of Plant Carbon Storage in Spatial Distribution

**Figure 5:**
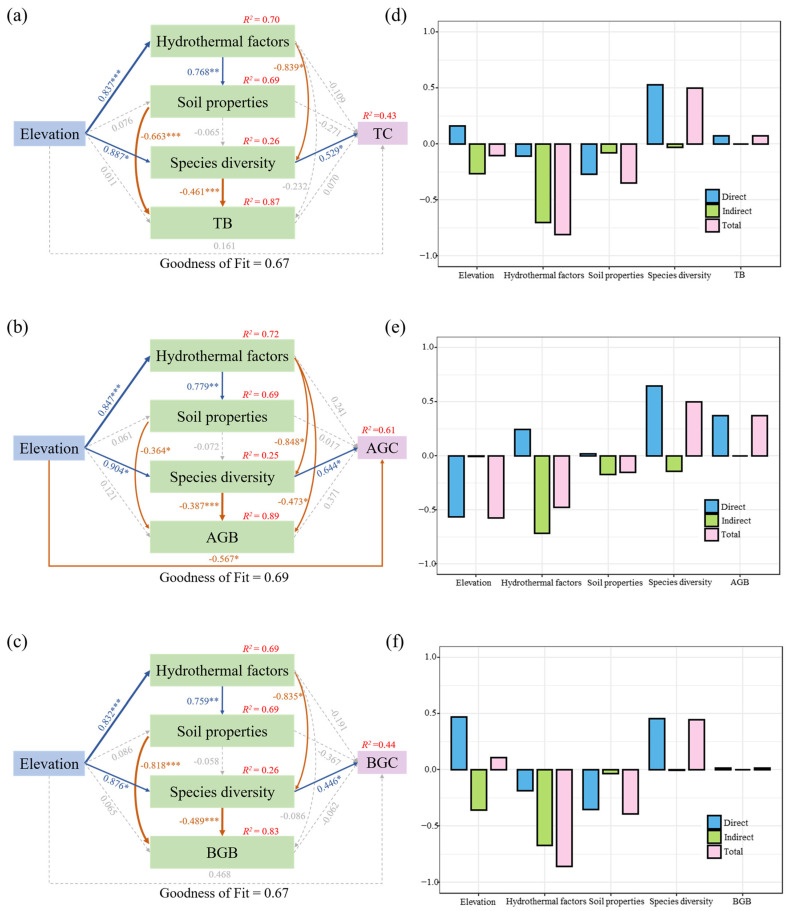
Partial least squares structural equation modeling (PLS-SEM) of the drivers that affect plant carbon storage. The path diagrams of (**a**–**c**) depict relationships and standardized path coefficients for the total (TC), aboveground (AGC), and belowground (BGC) carbon storage, respectively. The bar charts of (**d**–**f**) show the corresponding direct, indirect, and total effects, respectively, of each driver for the TC, AGC, and BGC. The TB, AGB, and BGB are the plant biomass indices. The species diversity includes the R, H′, H, and E. The soil properties comprise the SOC, TN, TP, COND, and pH. The hydrothermal factors include the AT, AH, ST, and SW. These indices are the same in Figure 4. Data were obtained from 160 quadrats from all of the SGs. The asterisks denote the significance levels (i.e., *: *p* < 0.05; **: *p* < 0.01; ***: *p* < 0.001). Blue solid arrows show significantly positive correlations, brown solid arrows show significantly negative correlations, and gray dotted arrows show non-significantly correlations (positive or negative, *p* > 0.05).

At the overall scale (Figure 5a,d), hydrothermal factors were the primary driver of the TC spatial distribution. These factors exerted their influence indirectly by significantly regulating the species diversity. Its total effect on the TC was −0.8107. The species diversity acted as a secondary driver, directly and significantly affecting the TC distribution (total effect = 0.4969, *p* < 0.05). The soil properties exhibited no significant effect on the TC (total effect = −0.3501). Elevation did not exert a significant direct effect on the TC, whereas it indirectly influenced the TC distribution by significantly affecting both the hydrothermal factors and species diversity. This resulted in a total effect of −0.1041. The total biomass (TB) also exhibited no significant direct effect on the TC, with a total standardized effect of 0.0697. We quantified the direct and indirect standardized effects of each latent variable, and we assessed their relative influence on the TC. The order of the direct effects of the latent variables on the TC, in descending order of magnitude, was as follows: species diversity > soil properties > elevation > hydrothermal factors > TB. In contrast, the order for the indirect effects was as follows: hydrothermal factors > elevation > soil properties > species diversity > TB.

For the aboveground part (Figure 5b,e), elevation served as the primary driver of the AGC spatial distribution, exerting a direct and significant effect (*p* < 0.05). It also indirectly influenced the AGC by significantly affecting both the hydrothermal factors and species diversity, yielding a total effect of −0.5746. Species diversity acted as a secondary driver, exerting a direct and significant effect on the AGC (total effect = 0.4997, *p* < 0.05). Hydrothermal factors indirectly influenced the AGC by regulating the species diversity (total effect = −0.4788). In contrast, both the aboveground biomass (AGB) and soil properties exhibited no significant effect on the AGC, with total effects of 0.3717 and −0.1546, respectively. Among these latent variables, the order of the direct effects on the AGC in descending order was as follows: species diversity > elevation > AGB > hydrothermal factors > soil properties. In contrast, the order of the indirect effects was as follows: hydrothermal factors > soil properties > species diversity > elevation > AGB. Among the drivers examined, elevation, hydrothermal factors, and species diversity significantly affected the AGC (*p* < 0.05) and served as important predictors of the plant carbon storage variation.

For the belowground part (Figure 5c,f), hydrothermal factors served as the primary drivers of the BGC spatial distribution. Species diversity acted as a secondary driver, and it exerted a direct and significant effect on the BGC (total effect = 0.4464, *p* < 0.05). In contrast, the soil properties exhibited no significant influence (total effect = −0.3931). Elevation indirectly influenced the BGC through hydrothermal factors and species diversity (total effect = 0.1082), while the belowground biomass (BGB) showed no significant effect (total effect = 0.0135). Among the latent variables, the order of the direct effects on the BGC, in descending order, was as follows: species diversity > elevation > soil properties > hydrothermal factors > BGB. Conversely, the order of the indirect effects was as follows: hydrothermal factors > elevation > soil properties > species diversity > BGB. Among the driving factors examined, the hydrothermal conditions and species diversity significantly affected the BGC (*p* < 0.05) and served as important predictors of the plant carbon storage variation.

## 3. Discussion

### 3.1. Elevational Distributions of the Carbon Storage in SG Plants

Based on the data from this study, the hypothesis that plant carbon storage in the SGs of the eastern Loess Plateau exhibits a non-linear distribution along the elevational gradient was validated (Figure 3). Elevation is a key topographic factor that regulates temperature and water distribution [33,34,35]. These hydrothermal changes subsequently alter the plant biomass [36,37], species composition [38], and soil physicochemical properties [39], thereby indirectly affecting plant carbon storage [40,41,42]. This study demonstrated that the plant carbon storage in the SGs of the eastern Loess Plateau exhibited a U-shaped distribution along the elevational gradient (Figure 3).

Previous studies have documented diverse elevational plant carbon storage patterns. For instance, an inverted U-shaped (humped) pattern was observed in the arid–warm river valleys of eastern Yunnan, China [43], and in the Himalayan region of Kuru, northwest India [44]. In addition, monotonically decreasing trends were reported for the Changbai Mountain forest in northeast China [45] and the Boralae Forest in western Ethiopia [46]. The results of this study demonstrated that the total, aboveground, and belowground plant carbon storage all exhibited a U-shaped distribution along the elevational gradient, with the minima occurring at intermediate elevations and high spatial variability across the transect (Figure 3). This pattern is primarily driven by environmental factors.

The mid-elevation zone lies in a transitional hydrothermal niche, where the combination of moisture and temperature conditions reaches a relatively unfavorable balance. Vegetation growth is constrained to some extent, which suppresses plant productivity and carbon accumulation, leading to a trough in carbon storage [44]. Moreover, compared to low-elevation areas, the air temperature in the mid-elevation zone declines but remains insufficiently low to completely inhibit microbial activity; the decomposition rate of organic matter is still relatively high [44]. The combined effects of reduced carbon input and sustained decomposition results in decreased carbon accumulation efficiency.

Simultaneously, in contrast, the relatively high carbon storage observed in low-elevation areas primarily results from the more favorable hydrothermal conditions [47]. The relatively higher temperatures in low-elevation regions, combined with moisture conditions sufficient to support the growth of drought-tolerant grasslands, promote vegetation productivity and consequently increase plant biomass. Moreover, the grassland type in this elevation zone is dominated by mountain steppe, which exhibits higher plant carbon storage than alpine meadows [45,48].

At high elevations, carbon storage rises again to relatively high levels. This phenomenon is primarily attributed to the low-temperature conditions at high altitudes. Although the growing season for plants in high-elevation areas is short, the supply of cloud and fog condensate formed under low-temperature conditions alleviates drought stress, preventing biomass in this region from becoming too low [44]. Meanwhile, in the alpine environment, microbial activity is greatly reduced due to low temperatures, leading to an extremely slow decomposition rate of litter and root residues, which allows organic carbon to accumulate over long periods. These two factors synergistically promote carbon sequestration, making this region a high-value area for carbon storage [49]. Moreover, the highly heterogeneous topography and spatially differentiated soil properties across the Loess Plateau further amplify the variability in carbon storage patterns, resulting in significant data dispersion around the overall U-shaped elevational trend [50,51]. These findings highlight the interactive effects of multi-scale environmental factors and human activities on carbon cycling in montane ecosystems, providing important insights into the formulation of targeted carbon management strategies in fragile ecosystems under conditions of global change.

The above- and belowground allocation pattern exhibited distinct elevational contrasts. A substantial disparity between the aboveground and belowground carbon storage existed at both low and high elevations and manifested as a pronounced belowground allocation bias. In contrast, the difference between the aboveground and belowground stocks was the smallest at mid-elevations (Figure 3). This divergence stems from contrasting environmental drivers. In low-elevation areas, water scarcity drives plants to enhance their root water uptake capacity by increasing carbon allocation to belowground organs, thereby maintaining water balance [52]. In high-elevation areas, low temperatures and short growing seasons constrain aboveground growth, while the cold climate concurrently inhibits organic matter decomposition. This promotes long-term carbon storage in belowground organs, resulting in a pronounced belowground allocation bias [44]. In mid-elevation regions, relatively favorable hydrothermal conditions prevail. However, intense anthropogenic pressures, such as grazing, drive plants to reduce the belowground carbon allocation while prioritizing aboveground growth and reproduction [52,53]. This trade-off results in a carbon allocation pattern where the disparity between the aboveground and belowground stocks was smaller than that at both the lower and higher elevations. This carbon allocation pattern reflected the adaptive trade-off between resource acquisition and reproductive strategies in plants across different altitudinal environments.

### 3.2. The Carbon Storage Distribution Among Different Plant Functional Groups in SGs

This study confirms that in the SGs of the eastern Loess Plateau, forbs and sedges are the dominant functional groups for carbon sequestration (Figure 2). Significant differences in carbon storage were observed among plants of different functional groups within the study area (Figure 2). The overall distribution pattern (forbs > sedges > grasses) primarily reflected differences in the life history strategies and resource acquisition capacities among these functional groups. Forbs, predominantly comprising broadleaf perennial or annual plants from families such as Asteraceae and Fabaceae, generally exhibit a high leaf area index and photosynthetic rate, facilitating rapid aboveground biomass accumulation during the growing season [54]. Additionally, biological nitrogen fixation in some leguminous forbs further enhances carbon assimilation, collectively contributing to their highest total carbon storage among the functional groups [54]. Despite the well-developed belowground rhizome systems of sedges, they achieve significantly lower total carbon storage than forbs. This is primarily due to their relatively short stature and constrained photosynthetic area [55].

The results of this study differ to some extent from those of previous studies. Previous studies have shown that the carbon storage of sedges is higher than that of forbs [16], whereas in this study, the carbon storage of forbs was higher than that of sedges. The main reason for this discrepancy lies in the differences in estimation methods: previous studies estimated carbon storage by multiplying biomass by a uniform carbon conversion coefficient of 0.45, whereas the present study calculated carbon storage based on the measured organic carbon content of plants from each functional group. Different functional groups differ in their degree of lignification and tissue chemical composition, and their actual carbon contents are not entirely consistent. Using a uniform coefficient may mask the differences in carbon accumulation among functional groups. By measuring actual carbon content, the characteristics of carbon storage in different functional groups can be reflected more accurately. In a cold subalpine environment, grasses exhibited the lowest area-based carbon storage among the three functional types. This can be attributed to their inherently slower growth rates coupled with rapid leaf turnover and higher tissue lignification [56].

Furthermore, the plant carbon allocation between aboveground and belowground compartments differed markedly among the functional groups (Figure 2). Specifically, grasses and forbs allocated a higher proportion of carbon to aboveground structures, whereas sedges exhibited a pronounced bias toward belowground investments. This divergence in allocation reflects the contrasting competitive strategies evolved by these groups to occupy distinct ecological niches. Sedges predominantly rely on extensive rhizome and root networks to integrate and store resources. This enhances their persistence and competitiveness in heterogeneous or disturbed environments [57]. In contrast, grasses and forbs primarily compete for light by investing in rapid growth and high aboveground biomass production [58].

### 3.3. Driving Factors of the Elevational Carbon Storage Distribution in SG Plants

This study confirms that the synergistic effect of hydrothermal conditions and species diversity is the dominant factor driving the elevational pattern of plant carbon storage in the SGs of the eastern Loess Plateau (Figure 5). The spatial distribution of plant carbon storage was co-regulated by a suite of biotic and abiotic factors in the SGs of the eastern Loess Plateau. Crucially, these drivers operate through distinct pathways and exhibit markedly different relative contributions to the overall pattern (Figure 5). A comprehensive analysis revealed that the hydrothermal factors exerted the most substantial influence on plant carbon storage, primarily through indirect pathways that shaped its spatial distribution [16]. Species diversity acted as the secondary driver, exerting a direct and significant effect (Figure 5). Elevation indirectly regulated plant carbon storage by significantly modulating both the hydrothermal conditions and species diversity (Figure 5). In contrast, the effects of both the soil properties and biomass were statistically non-significant. It was evident that carbon storage in subalpine grassland plants exhibited high sensitivity to environmental variability. In the context of global climate change, the environmental sensitivity of this carbon pool carries dual ecological implications. Its responsiveness to hydrothermal variations offers the potential to enhance regional grassland carbon sequestration; however, it also indicates a vulnerability to carbon loss under intensified environmental stress [38,39]. Consequently, the carbon sequestration dynamics in these SGs can serve as an effective bioindicator of regional climatic and environmental shifts, providing a valuable early-warning system [39,59]. Furthermore, it implies that the targeted management of key hydrothermal conditions could effectively enhance grassland carbon sequestration and ensure the long-term stable functioning of ecosystem carbon sinks.

The effects of hydrothermal factors on total carbon storage and belowground carbon storage are entirely indirectly mediated by species diversity (Figure 5). This phenomenon reflects intensified species competition. In areas with better hydrothermal conditions, species diversity is generally higher, but the increased species are mostly companion species or small-statured forbs, which contribute little directly to community carbon storage [16,56]. However, they compete with high-carbon-accumulating dominant species (such as tall forbs or sedges with well-developed root systems) for light, water, and nutrients, thereby inhibiting the growth and carbon accumulation of the dominant species.

Species diversity has a significant positive direct effect on total carbon storage, aboveground carbon storage, and belowground carbon storage (Figure 5). This finding is consistent with the results of Weiskopf [20]. In communities with higher species diversity, different species exhibit differences in resource utilization strategies, enabling them to more fully exploit environmental resources such as light, water, and nutrients through niche complementarity mechanisms [20]. Additionally, highly diverse communities are more likely to contain productive dominant species, which enhance the overall carbon accumulation capacity of the community through the selection effect [56].

Elevation indirectly influenced the spatial distribution of plant carbon storage by altering local climatic conditions, vegetation compositions, and soil microbial diversity [60]. Increasing elevations led to lower temperatures, altered precipitation regimes, and a higher solar radiation intensity [60]. These resulting hydrothermal conditions directly constrain plant photosynthetic and respiratory rates and influence soil nutrient availability, thereby imposing physiological limits on the carbon sink potential [61]. Concurrently, these environmental gradients act as filters that shape the vegetation community assembly, thereby promoting species diversity differentiation along the elevational transect [32,62,63]. Species diversity enhances carbon storage primarily by increasing the community resource-use efficiency and productivity through complementary and selection effects that in turn promote greater biomass accumulation [59,64]. Therefore, the spatial pattern of plant carbon storage is co-driven by abiotic environmental filtering and biotic processes along the elevational gradient. Within this dual-driver framework, abiotic factors set the environmental ceiling for carbon storage, while species diversity acted as the key biological pathway that directly enhanced the ecosystem functional efficiency to drive carbon sequestration. This mechanistic insight demonstrates that preserving highly diverse grassland communities and optimizing habitat conditions are effective pathways to enhancing the carbon sink function of SGs, providing essential scientific support for the management of grassland ecosystems under climate change.

### 3.4. The Limitations or Uncertainties in This Research

This study provides a foundation for comprehensively exploring and understanding the carbon storage distribution patterns along geographical gradients in high-elevation regions. However, it still has some limitations or uncertainties.

First, due to limitations regarding the study area (especially the fewer surveyed SGs) and survey time, this research primarily analyzed the summer vegetation structure and soil properties of the SGs in the eastern plateau. Additionally, there were certain human errors in operation when we extracted SG areas using ArcGIS 10.5 software, and some uncertainties in estimating plant carbon storage for entire area by extrapolating plot-level measurements to the landscape scale. These results can be verified better with more mountains with typical SGs and can be expanded to investigate the relative contributions of biotic and abiotic factors to plant carbon storage during different seasons.

Second, owing to plots being established in each SG in areas characterized by flat terrain, uniform vegetation, and no human disturbance, this study did not delve into the effects of human disturbance on plant and soil carbon storage. Those compelling and context-specific questions, tailored to the unique characteristics of SG plant communities on the Loess Plateau, e.g., interactions between soil erosion and carbon sequestration, will be better for providing a more systematic scientific basis for the ecological conservation and restoration of SGs in high-elevation regions.

## 4. Materials and Methods

### 4.1. Study Area

**Figure 6:**
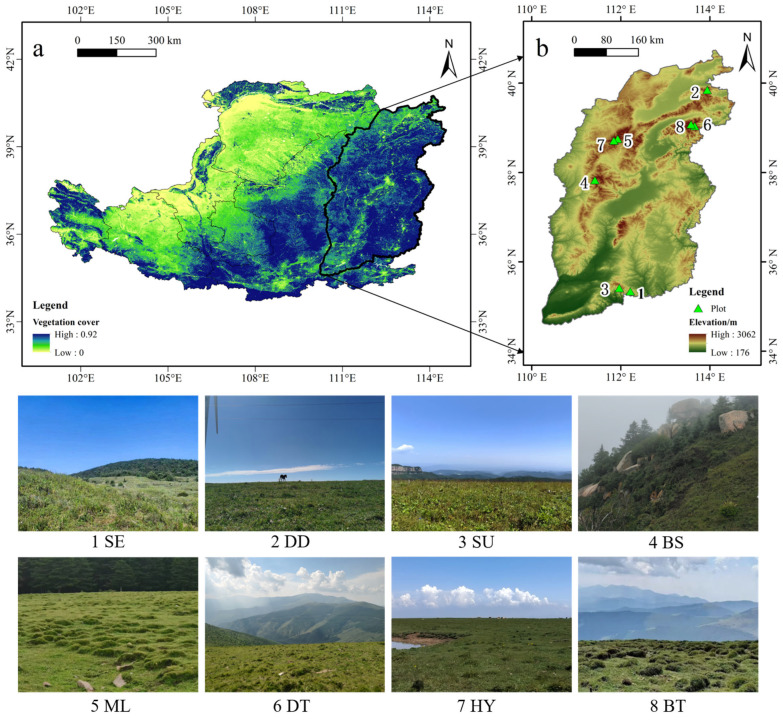
Location of the study site and representative plot photographs: the Loess Plateau (**a**) and Shanxi Province (**b**) of China. (**a**) shows the grassland cover in the Loess Plateau. (**b**) shows the topography of the Shanxi Province with mountains and basins. The eight photographs of the SGs correspond to the surveyed mountains in (**b**), with the abbreviations of SE, DD, SU, BS, ML, DT, HY, and BT representing the mountains in Table 3. The numbers in subfigure b are sequence number for surveyed mountains and are ordered with increased elevations.

The Loess Plateau possesses the most extensive global distribution of loess (Figure 6a). It exhibits extremely high ecological fragility due to sparse precipitation, intense evaporation, and severe soil erosion [16]. This makes ecological restoration in degraded areas exceptionally challenging. The east of the Loess Plateau (110°14′–114°33′ E, 34°34′–40°43′ N) is located in Shanxi Province, and it has a total area of 156,700 km^2^ (Figure 6b). The terrain within this region is complex and diverse, with mountains and hills accounting for 80.1% of the province’s total area. In addition, the average elevation exceeds 1500 m [16]. The region experiences a temperate continental monsoon climate that is characterized by an average annual temperature of 4–14 °C, an annual precipitation of 400–600 mm, and a frost-free period of 4–7 months [60]. This terrain and climatic regime foster typical SG vegetation growth. The SG vegetation covers an area of approximately 353,000 hm^2^ and is primarily distributed above the tree line in major mountain ranges, with elevations ranging from 1700 to 3000 m [56]. SG communities exhibit high species richness, with 172 plant species recorded [56]. These are predominantly cold-tolerant mesophytic perennial herbaceous plants that include families such as Poaceae, Asteraceae, Fabaceae, Cyperaceae, and Polygonaceae. Common species include *Carex lanceolata*, *Kobresia humilis*, *Saussurea japonica*, and *Polygonum viviparum*. The soil types are diverse, and they include loess soils, meadow soils, chestnut calcareous soils, brown earth, and saline–alkaline soils [61]. Among these, the primary SG soil types are mountain brown soils and subalpine meadow soils. The parent material is predominantly residual–colluvial deposits of metamorphic conglomerate and gneissic granite that is characterized by a high organic carbon density that exceeds 13 kg/m^2^ in some areas. The low temperatures in SGs allow for the development of seasonal permafrost in the soils of certain grasslands. This causes the formation of frost heaves on the soil surface that influence the spatial distribution of SG plants [62,63].

**Figure 7:**
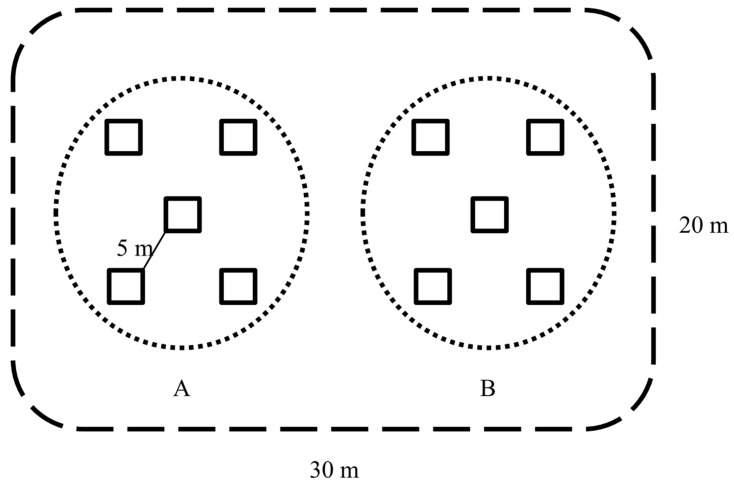
Experimental plot designs with sampling quadrats in 2023 and 2024. The surveyed SG plots were designed to be 600 m^2^. In each plot, the quadrats were sampled at a size of 1 m^2^ with a randomized block design (two blocks of A and B in total). In each block, five quadrats (at intervals of 5 m) were placed using a cross method in the SGs. In total, there were 20 quadrats for each SG (or mountain).

From July to August in 2023 and 2024, mountains with large SG areas were sequentially selected from the north to the south across the eastern plateau. These areas covered the Liuleng, Wutai, Lüliang, and Zhongtiao mountain systems with elevations greater than 1700 m. One well-developed SG was selected above the tree line on each mountain, and eight SGs were selected in total. The greatest difference among these SGs was 1232 m for elevation, 2.55° for longitude, and 4.43° for latitude (Table 3). The relatively smaller differences for longitude and latitude of surveyed SGs can avoid confounding site effects with elevation effects in data processing. These SG areas were examined in each year of the study. A plot with an area of 600 m^2^ was established in each SG in areas characterized by flat terrain, uniform vegetation, and no human disturbance (Figure 7). Randomized block designs with two blocks were then constructed on the vegetation of the plots. Five 1 m^2^ quadrats (with intervals of 5 m) were then placed in each plot using a cross method to survey the plant species diversity within the SG community. The specific sampling locations and related information are shown in Figure 6b and Table 3.

### 4.2. Plant Community Investigations

The plant community survey included the height, abundance, cover, and frequency for each plant species to calculate the species diversity indices. In addition, plant samples were collected by harvesting all individuals within each quadrat, and soil blocks were excavated separately to collect the belowground root systems [16]. Species with richness values of less than 10 were removed entirely, while species with richness values greater than 10 were sampled at a rate of 10 individuals per species for the laboratory analysis. The whole-plant samples were thoroughly rinsed in the laboratory. Subsequently, the processed aboveground stems and leaves, together with the collected live root samples, were dried in an oven at 80 °C. The dried samples were weighed using an electronic balance with a precision of 0.001 g to determine the aboveground and belowground biomass values. Species names and taxonomic information were obtained from the *Flora of China* (http://www.iplant.cn). The surveyed species in each SG were classified based on functional traits into the following three groups: grasses, sedges, and forbs [16,17] (Supplementary Table S1).

The plant materials were then ground into fine powders using a ball mill. The plant organic carbon (POC) concentration was determined via the potassium dichromate oxidation method with external heating according to the standard protocols described in the Principles and Techniques of Plant Physiology and Biochemistry Experiments [64]. The operating procedures were as follows: after the plant samples were pulverized by the ball mill and sieved, 0.0100–0.0200 g (accurate to 0.0001 g) was weighed and placed at the bottom of a hard-glass test tube. Then, 5.00 mL of 0.8000 mol·L^−1^ potassium dichromate standard solution and 5.00 mL of concentrated sulfuric acid were added successively. After shaking to mix, a bent-neck small funnel was inserted into the tube mouth. The test tubes were placed into an oil bath at 180–185 °C, and the oil bath temperature was maintained at 170–180 °C. Timing started when the solution began to boil. After boiling for 5 min, the test tubes were removed and allowed to cool slightly, and the external oil stains were wiped clean. The contents and residues in the test tubes were transferred completely into 250 mL conical flasks. The test tubes and small funnels were rinsed several times with distilled water, and the rinsing solutions were also transferred into the conical flasks, adjusting the total solution volume to 60–70 mL. Three to five drops of phenanthroline indicator were added, and the solution was titrated with 0.2000 mol·L^−1^ ferrous sulfate standard solution until the color changed from orange-yellow to blue-green and then abruptly to brick-red, which indicated the endpoint. The volume of ferrous sulfate consumed was recorded. A blank test was conducted simultaneously using quartz sand instead of the plant sample, following the same procedure. Since this method oxidizes only about 90% of the organic carbon, the measured results were multiplied by a correction factor of 1.1 to calculate the total organic carbon content of the plants. Each sample was measured three times, and the average value was taken.

### 4.3. Soil Property Measurements

Soil samples were collected using a 10 cm diameter soil auger at two depths of 0–10 cm and 10–20 cm. The samples were sealed and transported back to the laboratory for subsequent processing. The soil samples were air-dried in the laboratory at room temperature and then gently crushed using a rubber hammer. First, the visible impurities (e.g., stones and roots) were removed by sieving the soil through 40-mesh (0.42 mm aperture) and 60-mesh (0.25 mm aperture) sieves. Subsequently, a representative 30 g subsample was obtained by passing the sieved soil through an 80-mesh (0.18 mm aperture) sieve for the determination of the soil organic carbon (SOC), total nitrogen (TN), total phosphorus (TP), pH, and electrical conductivity (COND). The soil organic carbon (SOC) was determined using the potassium dichromate oxidation method with external heating. The total nitrogen (TN) was determined using the Kjeldahl method. The total phosphorus (TP) was determined using the molybdenum antimony colorimetric method. The pH was measured using potentiometry, and the electrical conductivity (COND) was determined using the conductivity meter method. The Soil Agro-chemical Analysis operating procedures were followed [65].

### 4.4. Hydrothermal Factor Monitoring

The hydrothermal factors (i.e., temperature and moisture) were monitored within each SG. Since plots were guardless, instruments could not be placed in the field for long time, and temperature and moisture were only measured during the vegetation investigation, with the corresponding data being in daytime hours (09:00–18:00). The instruments used in this study were a series of HOBO brand products produced by Onset Computer Company of America (Bourne, MA, USA). Three monitoring points, spaced 10 m apart, were arranged in an equilateral triangle within each plot. An air temperature and humidity sensor with a radiation shield (S-THB-M008) was installed at a height of 20 cm above the ground to measure air temperature (AT) and air humidity (AH), and temperature sensors (S-TMB-M006) and moisture sensors (S-SMD-M005) were inserted at depths of 10 cm and 20 cm to measure soil temperature (ST) and soil moisture (SW). All data on hydrothermal factors were automatically recorded at 1 min intervals by a data logger (H21-USB).

### 4.5. Data Analysis

#### 4.5.1. Calculation of the Species Diversity Indices

The Patrick, Shannon, Simpson, and Pielou indices were selected to calculate the plant species diversity indices within the local habitat of the SG communities. The equations are as follows:

(1) $$\begin{array}{c} \mathrm{IV}=\frac{rh+ra+rc+rf}{4}, \end{array}$$

(2) $$\begin{array}{c} R=S, \end{array}$$

(3) $$\begin{array}{c} H^{\prime }=1-\sum_{i=1}^{S}P_{i}^{2}, \end{array}$$

(4) $$\begin{array}{c} H=-\sum_{i=1}^{S}P_{i}\ln\left(P_{i}\right), \end{array}$$

(5) $$\begin{array}{c} E=\frac{H}{\ln\left(S\right)}, \end{array}$$

(6) $$\begin{array}{c} P_{i}=\frac{{IV}_{i}}{{IV}_{total}}. \end{array}$$

In these equations, IV is the importance value calculated as the sum of the relative height (rh), relative abundance (ra), relative frequency (rf), and relative cover (rc). Species diversity was assessed using the Patrick index (R), the Simpson index (H’), the Shannon index (H), and the Pielou index (E). Here, i denotes a plant species within a quadrat, and S denotes the total number of all plant species recorded across the quadrat.

#### 4.5.2. Plant Carbon Storage Estimations

The plant carbon storage of each SG for the entire area was calculated using a tiered approach at the plot scale. First, the carbon density ($PC{pool}_{i}$) of each plant component was determined based on its biomass and carbon content. The total plant carbon storage was then obtained by scaling the carbon density with the SG area. Finally, the total plant carbon storage was partitioned into the aboveground and belowground components, which were further disaggregated by plant functional groups. The specific equations and variable definitions are as follows:

(7) $$\begin{array}{c} PC{pool}_{i}={Biomass}_{i}\times C_{i}\%, \end{array}$$

(8) $$\begin{array}{c} C=S\times PC{pool}_{i}, \end{array}$$

(9) $$\begin{array}{c} TC=AGC+BGC, \end{array}$$

(10) $$\begin{array}{c} \mathrm{AGC}={\mathrm{AGC}}_{G}+{\mathrm{AGC}}_{S}+{\mathrm{AGC}}_{F}, \end{array}$$

(11) $$\begin{array}{c} \mathrm{BGC}={\mathrm{BGC}}_{G}+{\mathrm{BGC}}_{S}+{\mathrm{BGC}}_{F}. \end{array}$$

In these equations, i represents the plant components (e.g., aboveground parts, litter, and roots); $PC{pool}_{i}$ is the carbon density of each component i; [Biomass]_i_ is the biomass of each component i; $C_{i}\%$ is the carbon content of each component i; C is the total carbon stock of the subalpine grassland vegetation (in Tg or Mg, where 1 Mg = 106 g and 1 Tg = 1012 g); and S is the SG area.

In each SG, the plant aboveground carbon storage (AGC) was defined as the sum of the contributions from grasses (AGC_G_), sedges (AGC_S_), and forbs (AGC_F_); the plant belowground carbon storage (BGC) was similarly defined as the sum of the contributions from grasses (BGC_G_), sedges (BGC_S_), and forbs (BGC_F_); and the total plant carbon storage (TC) was defined as the sum of the AGC and BGC at the plot scale.

#### 4.5.3. SG Area Extraction

The area of each SG was extracted to calculate the plant carbon storage at the plot scale. ArcGIS 10.5 software was used to establish the spatial analysis framework by first loading 1:250,000 topographic maps and 1:1,000,000 vegetation maps of Shanxi Province as basemaps. A uniform geographic coordinate system (GCS_WGS_1984) was defined as the reference framework. Subsequently, raster basemaps were georeferenced to ensure precise spatial alignment between the raster pixels and vector data boundaries. A comma-separated values (CSV) table that contained the latitude, longitude, and elevation data for the eight sample mountain ranges was created. This table was added to the map, and a spatial point feature layer was generated from it using the “Display XY Data” tool.

In order to focus our analysis on the potential distribution zones that surrounded each sample mountain range and to optimize the computational efficiency, a 5 km radius buffer was created around each sampling location. The selection of 5 km as the radius of the buffer zone is mainly based on the objective scale of the spatial distribution characteristics of the sub-alpine grassland and the mountain natural geographical units in the study area, rather than the subjective setting. The alpine grassland in Shanxi is mostly concentrated on the top of the medium- and high-altitude mountain body and the gentle slope area. Affected by the elevation gradient, topographic cutting, and ditch and valley division, the continuous distribution range of sub-alpine grassland on a single mountain body is usually several kilometers. The 5 km buffer zone can cover the core distribution area of a single mountain more completely such that the analysis unit matches the actual habitat range of vegetation. This scale not only avoids insufficient habitat representation (a problem of too small a scope) but also prevents mixing into non-grassland ecosystems such as woodland, farmland, and villages (a problem of too large a scope), ensuring the purity and consistency of the research object. In addition, a radius of 5 km ensures that the buffer zones of different mountain samples do not overlap, avoids spatial nesting and information redundancy, and makes each unit relatively independent in terms of topography, climate, and vegetation conditions, thus more accurately reflecting the impact of the local environment on the carbon reserves of sub-alpine grassland plants.

After the 5 km radius buffer was created, the digital elevation model (DEM) and vegetation map were then batch-processed by clipping them to these buffers to ensure the complete spatial alignment of all subsequent data layers. Based on the defined lower elevational limit for the SGS (1720 m) in this study, the areas at or above this threshold were extracted from the corresponding DEM of each sample mountain range using the raster calculator tool. The extracted elevation zones were then overlaid with the vegetation map, and an intersection operation was performed to exclude non-grassland vegetation types (e.g., woodlands and shrublands). This procedure precisely delineated the subalpine grassland patches within each sample mountain range that satisfied both the elevational and vegetation-type criteria.

All data layers were uniformly projected into an Albers equal-area conic projection to ensure accuracy in the area calculations. The projection was defined based on the GCS_WGS_1984 geographic coordinate system, with a central meridian of 111°E, standard parallels at 36° N and 40° N, and a false easting and false northing of 0. These parameters were fine-tuned to align with the standard EPSG:3577 definition. The “Project” tool was used to batch-convert all of the data layers into this projected coordinate system. Following this projection transformation, the planar area of each grassland patch within the plots was calculated directly using the “Calculate Geometry” tool in ArcGIS. The results were output directly in hectares (hm^2^). The areas of the eight plots were then subjected to separate statistical analyses.

#### 4.5.4. Data Processing with Software

Data processing was conducted using a suite of complementary software applications. SPSS 27.0 was used to conduct a one-way analysis of variance (ANOVA) to test for significant differences in the plant carbon storage across the three functional groups (i.e., grasses, sedges, and forbs) among the different elevational sites. Additionally, a Pearson correlation analysis was performed to examine the relationships between the plant carbon storage and various biotic and abiotic factors. This facilitated the identification of key drivers. Origin 2021 software was used to generate an elevational trend plot of the plant carbon storage and a correlation heatmap between the carbon storage and the driving factors. The R 4.4.1 platform is a partial least squares structural equation model (PLS-SEM) constructed using the “plspm” package. The plant biomass and species diversity indices were specified as biotic latent variables, whereas the soil properties and hydrothermal factors were specified as abiotic latent variables. The plant carbon storage was designated as the dependent variable in the model. The path coefficients and the coefficient of determination (R^2^) were validated using a bootstrap procedure with 1000 iterations. Observable variables with factor loadings below 0.7 (specifically pH and the Pielou index E) were excluded from the model. The model goodness-of-fit was then assessed. Finally, the direct, indirect, and total standardized effects of each latent variable on the plant carbon storage were quantified to elucidate their driving pathways and relative contributions.

## 5. Conclusions

In the well-developed SGs of the eastern Loess Plateau, the plant carbon storage for entire area in each SG exhibited a U-shaped trend with elevation and was predominantly allocated to its belowground portions. However, this allocation pattern varied across different plant functional groups. Grasses and forbs tended to allocate carbon to their aboveground parts, while sedges adopted a belowground allocation pattern. In the SG communities, forbs and sedges were the primary contributors in carbon accumulation with greater carbon storage. The carbon storage in sedges and forbs was significantly influenced by plant species diversity and soil total phosphorus, while there was no significant correlation between grass carbon storage and any driving factors. Species diversity directly promoted plant carbon storage, and hydrothermal factors indirectly drove plant carbon storage by influencing species diversity. Therefore, the carbon storage of SG plants on the eastern plateau exhibited a nonlinear distribution pattern along an elevation gradient, with this elevational differentiation being primarily driven by the effects of hydrothermal factors and species diversity.

Within this dual-driver framework, abiotic factors set the environmental ceiling for carbon storage, while species diversity acted as the key biological pathway that directly enhanced the ecosystem functional efficiency to drive carbon sequestration. This insight demonstrates that preserving highly diverse grassland communities and optimizing habitat conditions are effective pathways to enhancing the carbon sink function of SGs, providing essential scientific support for the management of grassland ecosystems in the eastern Loess Plateau. Future researches should focus on the interactive effects of multi-scale environmental factors and human activities on carbon cycling in montane ecosystems, which can provide important insights into the formulation of targeted carbon management strategies in fragile ecosystems under climate change.

## Figures and Tables

**Table 1:** Descriptive statistics of the aboveground (AGC), belowground (BGC), and total (TC) carbon storage in SG plants. Values are provided in megagrams (Mg). Data was obtained from a total of 160 quadrats for all SGs in 2023 and 2024. The coefficient of variation was calculated with the division of the standard deviation to the mean. The detained data of the plant AGC, BGC and TC in each surveyed SG is shown in Supplementary Table S2. *: the TC was calculated for each SG at the plot scale, so the mean TC was averaged to the 8 SGs.

Carbon Storage (Mg)	Maximum	Minimum	Mean	Median	Coefficient of Variation
TC *	8174.53	1125.10	4485.12	5002.06	0.53
AGC	2903.23	323.54	1252.94	1094.90	0.61
BGC	6596.10	694.09	3232.18	3488.67	0.55

**Table 2:** The carbon storage statistics for the three major plant functional groups in SGs. Plant carbon storage was subdivided into aboveground (AGC), belowground (BGC), and total (TC) carbon storage. Values are provided in megagrams (Mg). Data was obtained from a total of 160 quadrats for all SGs in 2023 and 2024. The corresponding carbon storage of grasses, sedges, and forbs in each SG is shown in Supplementary Table S3.

Carbon Storage (Mg)		Grasses	Sedges	Forbs
AGC	Maximum	179.53	1495.67	2220.27
	Minimum	0.00	54.33	110.50
	Mean	57.75	490.39	704.80
	Median	20.49	420.72	632.04
BGC	Maximum	691.61	3987.01	3905.21
	Minimum	0.00	83.93	319.46
	Mean	95.72	1492.42	1644.05
	Median	37.77	1322.96	1537.54
TC	Maximum	871.14	4421.10	4740.47
	Minimum	0.00	138.25	452.55
	Mean	153.47	1982.81	2348.85
	Median	56.59	1987.77	2236.60

## Data Availability

The original contributions presented in this study are included in the article/Supplementary Materials. Further inquiries can be directed to the corresponding author.

## Supplementary Materials

The following supporting information can be downloaded at: https://www.mdpi.com/article/10.3390/plants15111696/s1, Table S1: Total plant species richness and species list by functional groups—grasses, sedges, and forbs—at the eight sampling SGs. Table S2: Plant carbon storage (TC, AGC, and BGC) of SGs at different mountains. Table S3: Carbon storage of different plant functional groups—grasses, sedges, and forbs—in SGs. TC, AGC, and BGC denote total, aboveground, and belowground carbon storage, respectively. Table S4: The F-values and degrees of freedom from the one-way ANOVAs in Figure 1 and Figure 3. Table S5: Summary of Pearson correlation analyses in Figure 4. Table S6: Curve estimations on relationships between elevation and total (TC), aboveground (AGC), and belowground (BGC) carbon storage, respectively.

## Abbreviations

The following abbreviations are used in this manuscript:

SGs	Subalpine grasslands
R	Patrick index
$H^{\prime }$	Simpson index
H	Shannon index
E	Pielou index
TC	Total plant carbon storage
AGC	Plant aboveground carbon storage
BGC	Plant belowground carbon storage
AGC_G_	Aboveground carbon storage of grasses
AGC_S_	Aboveground carbon storage of sedges
AGC_F_	Aboveground carbon storage of forbs
BGC_G_	Belowground carbon storage of grasses
BGC_S_	Belowground carbon storage of sedges
BGC_F_	Belowground carbon storage of forbs
SOC	Soil organic carbon
TN	Total nitrogen
TP	Total phosphorus
COND	Electrical conductivity
pH	Power of hydrogen
AT	Air temperature
AH	Air humidity
ST	Soil temperature
SW	Soil water content
TB	Total biomass
AGB	Aboveground biomass
BGB	Belowground biomass
SE	Shengwangping
DD	Diandingshan
SU	Shunwangping
BS	Bashuigou
ML	Maluncaoyuan
DT	Dongtai
HY	Heyeping
BT	Beitai
