# Supplementary material for: The Elevational Distribution Patterns and Driving Factors of Plant Carbon Storage Across Different Functional Groups in Subalpine Grasslands of the Eastern Loess Plateau, China
Source: Plants (Basel). 2026 May 30;15(11):1696. doi: 10.3390/plants15111696 (PMC13258829; doi:10.3390/plants15111696)
Supplement: Supplementary file 1 [file plants-15-01696-s001.zip › Supplementary Tables.pdf]

Supplementary Table S1. Total plant species richness and species list by functional groups—grasses, sedges, and forbs—at the eight sampling SGs. SE, DD, SU, BS, ML, DT, HY, and BT are the abbreviated names of the mountains, with the full names being listed in Table 1. The number of plant species in each SG is the total surveyed in 2023 and 2024, with repeated species being counted only once.

Mountain Name	Number of species	Grasses	Sedges	Forbs
SE	24	Calamagrostis epigeios, Elymus dahuricus	*Carex lanceolata*	*Geranium wilfordii, Sanguisorba officinalis, Descurainia sophia, Galium verum* var. *asiaticum, Vicia unijuga, Stellera chamaejasme, Saussurea japonica, Cenchrus echinatus, Fragaria vesca, Ranunculus japonicus, Leontopodium leontopodioides, Adenophora stenanthina, Chrysanthemum chanetii, Echinops sphaerocephalus, Adenophora capillaris, Anaphalis sinica, Dracocephalum rupestre, Delphinium grandiflorum, Potentilla discolor, Clematis florida, Plantago asiatica*
DD	23	Elymus dahuricus	*Kobresia capillifolia*	*Sanguisorba officinalis, Saussurea japonica, Descurainia sophia, Polygonum viviparum, Saussurea chingiana, Leontopodium leontopodioides, Dianthus chinensis, Halenia corniculata, Vicia unijuga, Chrysanthemum chanetii, Anaphalis sinica, Pedicularis* sp.*, Ligusticum jeholense, Oxytropis* sp.*, Stellaria media, Fragaria vesca, Delphinium grandiflorum, Geranium wilfordii, Stellera chamaejasme, Adenophora stenanthina, Galium verum var. asiaticum*
SU	25	Calamagrostis epigeios, Elymus dahuricus	*Carex lanceolata*	*Sanguisorba officinalis, Ligularia sibirica, Rumex acetosa, Geranium wilfordii, Fragaria vesca, Thalictrum alpinum, Adenophora stenanthina, Anaphalis sinica, Polygonum ajanense, Saposhnikovia divaricata, Leucanthemum vulgare, Halenia corniculata, Chrysanthemum lavandulifolium, Vicia*

				unijuga, Aconitum carmichaelii, Echinops sphaerocephalus, Dracocephalum rupestre, Gentiana macrophylla, Chrysanthemum chanetii, Delphinium grandiflorum, Plantago asiatica, Saussurea iodostegia
BS	14	Elymus dahuricus	*Carex lanceolata*, *Kobresia humilis*	*Viola collina, Taraxacum mongolicum, Geranium wilfordii, Fragaria vesca, Polygonum aviculare, Leontopodium leontopodioides, Plantago asiatica, Pedicularis* sp., *Trigonotis peduncularis, Potentilla multifida, Ligusticum jeholense*
ML	18	Elymus dahuricus	*Kobresia humilis*	*Polygonum viviparum, Ligusticum jeholense, Taraxacum mongolicum, Potentilla anserina, Leontopodium leontopodioides, Saussurea japonica, Thalictrum aquilegiifolium* var. *sibiricum, Rheum officinale, Ranunculus japonicus, Viola verecunda, Geranium wilfordii, Euphrasia pectinata, Plantago asiatica, Pedicularis* sp., *Aster tataricus, Oxytropis* sp.
DT	21	Elymus dahuricus	*Kobresia capillifolia*, *Kobresia pygmaea*, *Kobresia humilis*, *Carex lanceolata*	*Potentilla fragarioides, Thalictrum alpinum, Aster tataricus, Ligusticum jeholense, Taraxacum mongolicum, Polygonum viviparum, Gentiana scabra, Stellaria media, Oxytropis* sp., *Leontopodium leontopodioides, Anemone cathayensis, Saussurea japonica, Dianthus chinensis, Anaphalis nepalensis, Lactuca indica, Leibnitzia anandria*
HY	20	Elymus dahuricus	*Kobresia pygmaea*, *Kobresia humilis*, *Carex lanceolata*	*Polygonum viviparum, Thalictrum aquilegiifolium* var. *sibiricum, Potentilla fragarioides, Leontopodium leontopodioides, Saussurea japonica, Pedicularis* sp., *Taraxacum mongolicum, Oxytropis* sp., *Plantago asiatica, Rheum officinale, Geranium wilfordii, Sanguisorba officinalis, Euphrasia pectinata, Ligusticum jeholense, Potentilla multifida, Papaver nudicaule*
BT	19	Elymus	*Kobresia pygmaea*, *Kobresia*	*Potentilla fragarioides, Taraxacum mongolicum, Ligusticum*

	dahuricus	*humilis*, *Kobresia tibetica*, *Carex lanceolata*	*jeholense, Anemone cathayensis, Cicuta virosa, Papaver nudicaule, Aster tataricus, Leontopodium leontopodioides, Saussurea japonica, Polygonum viviparum, Stellaria media, Plantago asiatica, Thalictrum alpinum, Rhodiola rosea*

Supplementary Table S2. Plant carbon storage (TC, AGC, and BGC) of SGs at different mountains. Data are shown as mean ± standard deviation in Mgs. Data were derived from 20 quadrats in total for each mountain in 2023 and 2024. Aboveground (AGC), belowground (BGC), and total (TC) carbon storage were estimated from the corresponding biomasses of all plants within each SE, DD, SU, BS, ML, DT, HY, and BT. These are the abbreviated names of the mountains, with the full names being listed in Table 1.

Mountain name	AGC	BGC	TC
SE	2587.15±61.19	4267.84±790.35	6854.99±811.67
DD	2001.89±159.20	4194.25±206.98	6196.14±296.95
SU	594.44±39.75	1046.78±90.44	1641.22±128.77
BS	552.52±70.70	931.47±93.89	1484.00±158.13
ML	346.24±22.62	1545.84±126.49	1892.08±118.15
DT	1075.44±40.58	2907.06±216.95	3982.51±255.62
HY	1301.46±50.88	4914.98±150.31	6216.44±197.20
BT	1300.02±113.80	5667.22±58.89	6967.23±154.16

Supplementary Table S3. Carbon storage of different plant functional groups—grasses, sedges, and forbs—in SGs. TC, AGC, and BGC denote total, aboveground, and belowground carbon storage, respectively. Values are shown as mean ± standard deviation in Mgs. Data were derived from 20 quadrats in total for each mountain in 2023 and 2024. The full names of the mountain abbreviations (SE, DD, SU, BS, ML, DT, HY, and BT) are provided in Table 1.

Carbon storage (Mg)	Mountain name	Grasses	Sedges	Forbs
TC	SE	307.14±30.65	3177.10±762.48	3399.92±275.83
	DD	27.85±19.09	2034.06±1195.95	4141.56±569.69
	SU	111.52±66.53	471.98±231.44	1051.02±278.92
	BS	280.17±102.35	242.54±107.62	1638.81±494.00
	ML	38.23±5.32	1291.54±169.87	560.57±95.05
	DT	24.48±21.69	3218.93±643.53	732.14±130.72
	HY	19.77±34.24	2138.55+410.90	4050.51±166.96
	BT	418.57±393.24	3287.79±1001.67	3216.25±1330.97
AGC	SE	153.93±9.67	1201.23±258.56	1231.16±208.76
	DD	10.31±8.54	481.37±164.46	1518.3±645
	SU	54.05±45.66	264.92±123.79	274.33±47.17
	BS	124.69±36.22	107.6±70.02	583.23±159.02
	ML	17.57±3.44	180.12±19.19	148.55±58.53
	DT	8.70±8.01	791.35±172.60	272.90±51.08
	HY	6.48±11.23	442.17±104.73	850.59±91.05
	BT	86.25±81.42	454.39±59.85	759.34±261.53
BGC	SE	153.22±21.29	1975.87±667.72	2168.75±93.69
	DD	17.54±10.77	1552.69±1032.54	2623.26±471.20
	SU	57.47±21.59	207.06±108.69	776.69±237.28
	BS	155.47±67.68	134.95±44.57	1055.58±336.16
	ML	20.65±3.05	1111.42±165.82	412.02±89.01
	DT	15.78±13.79	2427.58±519.59	459.24±130.41
	HY	13.28±23.01	1696.38±306.18	3199.93±78.07
	BT	332.32±311.92	2833.39±1031.66	2456.91±1256.99

Supplementary Table S4. The F-values and degrees of freedom from the one-way ANOVAs in Figures 1 and 3. TC, AGC, and BGC denote total, aboveground, and belowground carbon storage, respectively. AGC_G, AGC_S, and AGC_F refer to the AGC for grasses, sedges, and forbs, respectively. BGC_G, BGC_S, and BGC_F refer to the BGC for grasses, sedges, and forbs, respectively.

Carbon storage	F-values (degrees of freedom)	Significance
TC	$F(7, 152)=544.92$	$P<0.001$
TC_G	$F(7, 152)=23.26$	$P<0.001$
TC_S	$F(7, 152)=67.94$	$P<0.001$
TC_F	$F(7, 152)=110.01$	$P<0.001$
AGC	$F(7, 152)=1762.40$	$P<0.001$
AGC_G	$F(7, 152)=43.16$	$P<0.001$
AGC_S	$F(7, 152)=128.54$	$P<0.001$
AGC_F	$F(7, 152)=72.30$	$P<0.001$
BGC	$F(7, 152)=701.94$	$P<0.001$
BGC_G	$F(7, 152)=19.68$	$P<0.001$
BGC_S	$F(7, 152)=54.37$	$P<0.001$
BGC_F	$F(7, 152)=94.74$	$P<0.001$

Supplementary Table S5. Summary of Pearson correlation analyses in Figure 4. Values are correlation coefficients (r). Asterisks indicate statistically significant correlations (*: $P < 0.05$ and **: $P < 0.01$). These abbreviations are the same as in Figure 4.

	TB	AGB	BGB	R	H′	H	E	SOC	TN	TP	COND	pH	AT	AH	ST	SW
TC	0.064	0.105	0.038	0.701**	0.293	0.503*	−0.010	−0.036	−0.240	−0.730**	−0.173	0.312	0.133	−0.433*	0.342	−0.175
TC_G	0.105	0.176	0.057	−0.195	−0.153	−0.241	−0.007	0.011	0.124	−0.164	0.166	−0.070	0.082	0.064	0.330	−0.133
TC_S	0.102	0.113	0.092	0.629**	0.234	0.404	−0.222	0.084	−0.156	−0.647	−0.096	0.112	0.079	−0.298	0.178	0.014
TC_F	−0.001	0.043	−0.027	0.576**	0.275	0.467*	0.175	−0.129	−0.256	−0.560	−0.208	0.398	0.129	−0.427	0.336	−0.266
AGC	0.339	0.447*	0.261	0.557**	0.149	0.351	−0.109	−0.328	−0.432	−0.821	−0.317	0.395	0.510*	−0.557	0.680**	−0.315
AGC_G	0.377	0.490*	0.294	−0.320	−0.351	−0.394	−0.190	−0.219	−0.025	−0.248	0.070	−0.100	0.401	0.024	0.605**	−0.175
AGC_S	0.509*	0.594**	0.438*	0.415*	−0.004	0.137	−0.397	−0.311	−0.428	−0.824	−0.394	0.063	0.623**	−0.518	0.561**	−0.218
AGC_F	0.098	0.184	0.042	0.571**	0.266	0.471*	0.141	−0.240	−0.336	−0.604	−0.201	0.551**	0.268	−0.463	0.537**	−0.291
BGC	−0.060	−0.053	−0.062	0.693**	0.326	0.518**	0.033	0.093	−0.134	−0.619	−0.094	0.245	−0.042	−0.337	0.163	−0.098
BGC_G	−0.024	0.024	−0.052	−0.124	−0.053	−0.154	0.073	0.111	0.179	−0.113	0.193	−0.050	−0.064	0.076	0.182	−0.104
BGC_S	−0.048	−0.064	−0.037	0.631**	0.288	0.450*	−0.137	0.209	−0.045	−0.513	0.017	0.116	−0.116	−0.189	0.028	0.091
BGC_F	−0.046	−0.026	−0.056	0.517**	0.249	0.414*	0.172	−0.064	−0.192	−0.480	−0.188	0.284	0.052	−0.365	0.207	−0.226

Supplementary Table S6. Curve estimations on relationships between elevation and total (TC), aboveground (AGC), and belowground (BGC) carbon storage, respectively. The common models of linear, quadratic, logarithmic, power, and exponential are selected for curve estimations in the SPSS software. The number is 8 for all elevations, so values of AGC, BGC, and TC are averaged for each elevation. Data are in Supplementary Table S2.

Model	AGC		BGC		TC	
	R square	Significance	*R* square	Significance	*R* square	Significance
Quadratic	0.603	0.099	0.488	0.188	0.466	0.208
Logarithmic	0.404	0.09	0.004	0.888	0.024	0.715
Linear	0.356	0.119	0.014	0.781	0.01	0.817
Power	0.206	0.258	0	0.966	0.013	0.789
Exponential	0.172	0.306	0.006	0.86	0.003	0.89
